# 

**Published:** 1864-11

**Authors:** 


### DENTAL REGISTER

### OF THE WEST.

EDITED BY

J. TAFT AND GEO. WATT.

NOVEMBER,—1864.

**MONTHLY:**
Three Dollars per Annum, in advance.

CINCINNATI:
J. TAFT, PUBLISHER.

